# Hysteroscopic Findings and Operative Treatment: All at Once?

**DOI:** 10.3390/jcm12134232

**Published:** 2023-06-23

**Authors:** Valentina D’Urso, Ferdinando Antonio Gulino, Giosuè Giordano Incognito, Monia Cimino, Valentina Dilisi, Alessandra Di Stefano, Marianna Gulisano, Francesco Cannone, Stella Capriglione, Marco Palumbo

**Affiliations:** 1 Department of General Surgery and Medical Surgical Specialties, University of Catania, 95123 Catania, Italy; valentinadursocs@gmail.com (V.D.); giordanoincognito@gmail.com (G.G.I.); moniacim93@gmail.com (M.C.); dilisi.valentina92@gmail.com (V.D.); alessandradist@hotmail.it (A.D.S.); m.gulisano92@gmail.com (M.G.); mpalumbo@unict.it (M.P.); 2 Department of Obstetrics and Gynaecology, Azienda di Rilievo Nazionale e di Alta Specializzazione (ARNAS) Garibaldi Nesima, 95124 Catania, Italy; francesco.cannone@hotmail.it; 3 Department of Obstetrics and Gynecology, Ospedale “Santa Maria Alla Gruccia” Piazza del Volontariato 2, 52025 Montevarchi, Italy; scapriglione2016@gmail.com

**Keywords:** hysteroscopy, endometrial polyp, intrauterine adhesion, myoma, metroplasty

## Abstract

Hysteroscopy is considered not only a diagnostic instrument but also a therapeutic tool for many uterine pathologies. In the early 1990s, advances in technology and techniques made hysteroscopy less painful and invasive, allowing to increase in the number of gynecological procedures performed in an ambulatory setting without significant patient discomfort and with potentially significant cost savings. This is the so-called “office hysteroscopy” or “see-and-treat hysteroscopy”, whose spread has permitted the decrease of the number of procedures performed in the operating room with the benefit of obviating the need for anesthesia and dilatation of the cervical canal.

## 1. Introduction

Hysteroscopy (from the Greek term “hysteros”, uterus and “scopy”, to look) represents the gold standard for the evaluation of the uterine cavity permitting to bypass the significant limits of dilatation and curettage (D&C) [1]. D&C, typically performed under general anesthesia, is a blind technique associated with many limitations, including a high percentage of false negatives, because of its poor ability to discriminate focal intrauterine lesions [2]; incomplete removal of tissue, resulting in the persistence of underlying conditions or the need for additional procedures; high risks and potential complications, including infection, bleeding and uterine perforation [3]. Further progress in hysteroscopic practice, developed in the late 1990s, was represented by the “see-and-treat hysteroscopy” or outpatient operative hysteroscopy. This practice introduced the concept of a single procedure perfectly integrating the operative part in the diagnostic work-up. With the clear visualization provided by the hysteroscope, it allows precise targeting and treating of specific uterine conditions, reducing the risk of damage to surrounding healthy tissue and improving the effectiveness of the treatment. This was made possible using the advent of small-diameter scopes with continuous flow systems and with a 5 Fr operative channel for the use of mechanical instruments (e.g., scissors, biopsy cups, graspers, and corkscrews) [4]. Moreover, in 1997, the introduction of a versatile electrosurgical bipolar system, the Versapoint (Gynecare, Ethicon, NJ, USA) allowed an increasing number of pathologies that could be treated in office operative hysteroscopy reducing the use of resectoscopes and operating rooms to a smaller number of specific cases. Versapoint’s electrodes, designed for cutting, coagulation, vaporization, and ablation, allow for efficient and effective removal of tissue within the uterine cavity. The energy can be adjusted to achieve different surgical goals. For example, lower power settings are used for coagulation and hemostasis, while higher power settings are employed for cutting or vaporizing tissue. Moreover, it utilizes electrosurgical energy to simultaneously cut and coagulate tissue, resulting in reduced blood loss during the procedure. This can lead to improved visualization of the surgical field and decreased risk of excessive bleeding, ultimately enhancing patient safety [5]. In 2000, Lindheim et al. showed that operative procedures were successfully performed in 97% of cases with mechanical instruments and bipolar electrodes among a group of self-selected patients [6]. In 2002, Bettocchi et al. reviewed the benefits of the new 5 Fr bipolar electrosurgical equipment in the treatment of large benign intrauterine pathologies such as the collection of endometrial biopsies, the uterine adhesiolysis, as well as the treatment of polyps and myomas smaller than 1.5 cm, with excellent patient tolerance [7]. However, many procedures can be performed also with 5 Fr cold scissors and grasper, reducing the risk of visceral injuries (like uterine perforation or thermal damage to normal endometrium). In 2010, Di Spiezio Sardo et al. demonstrated that even other uncommon gynecological pathologic conditions could be treated safely and effectively in an office operative hysteroscopy setting, such as sterilization, emptying of hematometra, vaginal lesions (longitudinal septum, fibroepithelial polyp, vaginal endometriosis), uterine cystic neoformations, ablation of cervical stumps [8] and removal of uterovaginal packing [7,9]. Office hysteroscopy is a minimally invasive procedure that often requires only local or regional anesthesia, reducing exposure to general anesthesia. Thus, it is well appreciated by patients that avoid the inconvenience of visiting the operating room and the consequent risks of undergoing anesthesia. This is particularly beneficial for patients who may have contraindications; they can resume normal activities immediately after the procedure using only anti-inflammatory drugs (NSAID) [10]. There are only a few contraindications to hysteroscopy. It is typically not performed during pregnancy, especially during the first trimester, because it carries a risk of potential harm to the developing fetus. If there is a suspected pregnancy or if the patient is uncertain about their pregnancy status, a pregnancy test is usually recommended before proceeding with hysteroscopy. If a patient has an active pelvic infection, such as pelvic inflammatory disease (PID) or uterine infection, performing a hysteroscopy may exacerbate the infection or spread it further. In such cases, the infection should be treated and resolved before considering hysteroscopy. Hysteroscopy involves the use of instruments and sometimes the removal of tissue, which can cause bleeding. If a patient has a bleeding disorder or is currently experiencing uncontrolled vaginal bleeding, hysteroscopy may not be recommended until the bleeding is under control. If a patient has severe cervical stenosis, it may be challenging or not feasible to perform a hysteroscopy. In such cases, alternative approaches or further evaluation may be necessary. Uterine perforation is a rare but potential complication of hysteroscopy, where the instrument inadvertently punctures the uterine wall. If a patient has a known or suspected uterine perforation, hysteroscopy may be contraindicated due to the increased risk of complications. Certain severe medical conditions, such as uncontrolled hypertension, heart disease, or respiratory compromise, may increase the risks associated with hysteroscopy. In such cases, the procedure may need to be performed in a hospital setting with appropriate monitoring and support. Although diagnostic hysteroscopy is feasible in patients with endometrial cancer [11,12,13], exophytic-type cervical cancer is contraindicated for the risk of hemorrhage [14,15]. Appropriate equipment, proper training, and knowledge are sufficient to practice safe outpatient operative hysteroscopy. Practitioners must have the proper skills and expertise, mandatory to perform hysteroscopy [16]. Patient information must be provided before surgery and informed consent must be signed by the patient. Diagnostic hysteroscopy and, if necessary, biopsy or even surgery should be performed in a well-equipped operating room to ensure patient safety and privacy [15]. Ajmi et al. [17] conducted an observational study showing that second-generation endometrial ablation is an effective management option for heavy menstrual bleeding. The outpatient procedure is associated with a short hospital stay and quick recovery; it avoids general anesthesia and its complications and is highly acceptable to patients. Common office procedures include diagnostic hysteroscopy, endometrial biopsy with direct hysteroscopic visualization, endometrial polypectomy, lysis of intrauterine adhesions (IUA), resection of small submucous leiomyomas, placement of permanent contraceptive implants, removal of foreign objects or dislodged intrauterine devices, removal of retained products of conception, uterine metroplasty, and treatment of some obstetric conditions, such as endogenic cesarean scar pregnancy [7,18,19].

## 2. Hysteroscopic Procedures

### 2.1. Polypectomy

Endometrial polyps are localized hyperplastic overgrowths of endometrial glands and stroma. They are relatively common, especially in the reproductive years. They can vary in size, ranging from a few millimeters to a few centimeters, and they can occur as single polyps or multiple polyps. The exact cause of uterine polyps is not fully understood. However, hormonal imbalances, particularly high levels of estrogen, are believed to play a role in their development. They are often asymptomatic and are discovered incidentally during routine pelvic exams or imaging studies. However, some can also cause abnormal uterine bleeding (such as heavy or prolonged menstrual periods, irregular bleeding, or postmenopausal bleeding), pelvic pain, or infertility [20]. Removing the polyps can alleviate these symptoms and improve fertility outcomes. These lesions are usually benign; however, a small minority may have atypical or malignant features [21]. Outpatient hysteroscopic resection with direct visualization represents the optimal treatment modality for endometrial polyps, and the technique depends on the size of the polyp: small polyps (less than 0.5 cm) should be removed using a 5 Fr mechanical instrument such as sharp scissors and/or crocodile forceps; larger polyps (up to 2 cm) can be removed using bipolar energy such as a 15 Fr office resectoscope or the Versapoint Twizzle electrode; very large polyps or general polyposis are preferentially treated with a shaving system. It has been demonstrated that with new pump systems, shaving instruments are a safe and easy-to-learn option allowing for fast removal of soft tissue [22]. It is non-inferior to inpatient polypectomy under general anesthesia in the outcomes of improvement of bleeding as well as feasibility and acceptability of the procedure [23]. Outpatient polypectomy has also been found to be more cost-effective than inpatient polypectomy when followed-up 6 and 12 months after the procedure [24]. Hysteroscopic polypectomy has low complication and recurrence rates, and it is cost-effective. Moreover, it is technically feasible for practicing gynecologists who do not need much training [25,26].

### 2.2. Hysteroscopic Lysis of Intrauterine Adhesions

IUA, also known as uterine synechiae or Asherman syndrome, are bands of scar tissue that form inside the uterus. These adhesions can range from thin strands to thick bands and can occur in various locations within the uterine cavity. They are a consequence of the damage of the basal layer of the endometrium that can be caused by many factors, resulting in a fibrotic change and adhesion formation within the uterine cavity and the cervical canal. Uterine surgeries, such as D&C, myomectomy, or intrauterine surgery to control bleeding after childbirth can lead to adhesion formation. Infections within the uterine cavity, such as endometritis or tuberculosis, can cause inflammation and subsequent adhesion formation. Less common causes include radiation therapy, prolonged intrauterine device (IUD) use, or severe endometrial damage from pregnancy complications like placental abruption. They can lead to various reproductive health issues and may affect a woman’s fertility [20]. IUA can cause a variety of symptoms: they can interfere with the normal shedding of the uterine lining during menstruation, leading to light or absent periods; they can disrupt the implantation of a fertilized egg or cause a miscarriage by altering the shape and function of the uterine cavity; in some cases, intrauterine adhesions may cause intermittent or chronic pelvic pain. Hysteroscopy is considered the gold standard for the diagnosis of intrauterine synechiae because it allows the characterization of the location, extent, and type of adhesive disease [27]. Hysteroscopy is also the most effective therapy with 95% of women achieving successful restoration of menses and an overall risk of IUA recurrence of less than 30% [28,29]. Hysteroscopic adhesiolysis is associated with improvements in fertility and reproductive outcomes with an overall conception rate as high as 48% [30]; in particular, it is better to avoid the use of electrosurgery, because of the cumulative negative effect on pregnancy outcomes compared with adhesiolysis without application of energy (pregnancy rate of 16% vs. 29% in patients with two or more previous procedures) [31]. In most women with IUA, office-based adhesiolysis can be offered successfully with the use of hysteroscopic scissors, with nearly 90% of cases using preoperative NSAIDs alone for analgesia [29]. In patients with moderate to severe IUA, hysteroscopic adhesiolysis is associated with a high risk of uterine perforation, with rates of 3% to 5% [29], but avoiding electrosurgery reduces the risk of thermal injury to surrounding pelvic structures if perforation occurs. In the postoperative phase, there is a high risk of recurrence of IUA, and multiple strategies may be used to prevent this condition, associated with a second-look hysteroscopy performed within 2 weeks after the adhesiolysis to lyse any new synechiae avoiding becoming dense [32]. Hyaluronic acid has been used in antiadhesive gels for the management of Asherman syndrome and its introduction was thought to promote the separation of opposing endometrial surfaces [33]. Cyclic estrogen administered on a schedule that mimics the physiological menstrual cycle has been thought to stimulate endometrial regeneration, increase endometrial thickness, and possibly prevent recurrent or postoperative adhesions in women undergoing operative hysteroscopy [31]. Recent studies have utilized oral estradiol ranging from 2 to 6 mg daily [34,35]. Hysteroscopic cold scissors are more efficient in preventing intrauterine adhesion recurrence, increasing the menstrual flow, reducing intraoperative blood loss, and shortening the operation time [36]. The effectiveness rate of this procedure is approximately 90% for complete resolution of IUA when assessed at follow-up hysteroscopy 4 weeks later [30].

### 2.3. Hysteroscopic Myomectomy

Uterine leiomyomas, commonly known as uterine fibroids or simply fibroids, are the most common type of benign tumor in women. The exact cause of uterine fibroids is not fully understood. However, they are thought to develop from abnormal growth of uterine muscle cells. Hormonal factors, particularly estrogen and progesterone, play a role in their growth and development. They have a varied presentation depending largely on their size, number, and location. Though most fibroids are asymptomatic, approximately 30% of them cause abnormal uterine bleeding, anemia, pelvic pain or pressure, frequent urination, difficulty emptying the bladder, constipation, backache, and, in some cases, reproductive problems such as infertility [20] or recurrent miscarriages. The International Federation of Obstetrics and Gynecology (FIGO) published one of the most used classification systems, which describes type 0 lesions as completely within the endometrial cavity and type I lesions as those that extend <50% into the myometrium [37]. Malignancy is very rare, with a reported incidence as low as 0.1% for resected myomas [38]. Myomectomy is the most frequent intervention that sometimes cannot be transferred to the outpatient or ambulatory operating room. Management of leiomyomas should be tailored to the size and location of fibroids, patient’s age, symptoms, desire to maintain fertility, access to treatment, and the experience of the physician.

- Small type 0 submucous myomas with a diameter of less than 1 cm can be hysteroscopically removed using 5 Fr hysteroscopic scissors and tenaculum, without anesthesia, using a well-tolerated technique similar to polypectomy taking less than 10 min.
- For type 1, 2 and 3 myomas presenting an important intramural proportion, first, a gentle separation of the myoma from the capsule using mechanical instruments (scissors, grasping forceps) and a bipolar needle to pinpoint coagulation of the vessels is required. This step avoids myometrial stimulation or damage of the surrounding healthy myometrium, plus it coagulates important afferent and efferent vessels before a shaping procedure. After slicing the myoma, either a shaver, a 15 Fr office resectoscope, or a bipolar needle such as the Versapoint Twizzle electrode can be used. The use of miniaturized Office Hysteroscopes (Office Preparation of Partially Intramural Myomas: OPPIuM) represents a new ambulatory surgical technique for large (up to 1.5 cm) submucous myomas with partially intramural development (G1 and G2). This tool facilitates the subsequent resectoscopic removal under general anesthesia [39]. This technique consists of the incision of the endometrial mucosa and the pseudo-capsule covering the myoma allowing to push the myoma into the uterine cavity by the myometrial fibers. Distension fluids differ in viscosity, tonicity, and electrolyte status. Normal saline is a low-viscosity, isotonic solution with electrolytes, and it is preferred with bipolar electrocautery and mechanical instruments. In contrast, electrolyte-free fluids including hypotonic (1.5% glycine and 3% sorbitol) and isotonic solutions (5% mannitol) are used with monopolar electrocautery. Excessive absorption of hypotonic electrolyte-free solutions can lead to hypoosmolarity, hyponatremia, heart failure, pulmonary edema, cerebral edema, and hypotonic encephalopathy, while isotonic electrolyte-free fluids can cause hyponatremia. Excessive absorption of normal saline is associated with volume overload, pulmonary edema, and heart failure. Fluid absorption can be reduced by pre-operative treatment with GnRH agonists and intraoperative injection of vasopressin. If excessive absorption of hypotonic solution occurs, the patient’s serum electrolytes should be evaluated for volume overload. Asymptomatic hyponatremia can be managed by treatment with fluid restriction and monitoring urine output. Symptomatic hyponatremia requires an infusion of a 3% sodium chloride. Fluid overload from normal saline can be treated with fluid restriction. Finally, intravenous furosemide administration is indicated in the case of pulmonary edema [40]. It is paramount to recognize the critical importance of a thorough and accurate diagnosis before performing an operative hysteroscopy as a part of a “see-and-treat” procedure. This step is particularly crucial when significant uterine pathologies such as large myomas are presented. An accurate diagnosis not only facilitates therapeutic success but also serves as a crucial deterrent to potential complications. It is also necessary to highlight that even though operative hysteroscopy is a minimally invasive approach, the need for a high level of surgical and clinical experience on the part of the operator cannot be understated. The combination of a precise diagnosis and the operator’s proficiency ensures optimal patient outcomes and minimizes risks.

### 2.4. Hysteroscopic Metroplasty

Congenital uterine anomalies, also known as congenital uterine abnormalities or uterine malformations, refer to structural abnormalities of the uterus that develop during fetal development. These anomalies occur due to abnormal fusion or development of the Müllerian ducts, thought to occur in 5.5% of the general female population [41]. There are various types of congenital uterine anomalies. A uterine septum occurs when a wall or partition divides the uterine cavity partially or completely. It is one of the most common uterine anomalies. Uterine didelphys, also known as a double uterus, is characterized by the presence of two separate uterine cavities, each with its cervix. A unicornuate uterus is a condition where one-half of the uterus does not develop properly, resulting in a smaller uterus with only one fallopian tube. A bicornuate uterus is characterized by a heart-shaped uterus, with a deep indentation in the middle. An arcuate uterus is a mild form of uterine anomaly where there is a slight indentation or dip in the top of the uterus. The presence of congenital uterine anomalies can lead to various symptoms and complications. These anomalies are often associated with poor reproductive outcomes, such as implantation failure, cervical insufficiency [42], recurrent miscarriage, preterm labor [43], and abnormal fetal presentation, or can be asymptomatic [44]. Some women may experience abnormal menstrual bleeding, including heavy or prolonged periods, or may have difficulty using menstrual products due to the altered uterine shape. Congenital uterine anomalies are typically diagnosed through imaging studies, such as ultrasound (especially three-dimensional ultrasound), magnetic resonance imaging (MRI), or hysteroscopy. However, accurate diagnosis remains challenging [45] and misclassification of an arcuate uterus as septate at the time of hysteroscopy can lead to unnecessary treatment [46,47]. Class 1 dysmorphic uterus and Class 2 septate uterus are the most accessible surgeries for an ambulatory approach. Office hysteroscopic removal of the uterine septum is a simple and safe procedure and it has been suggested that it is associated with an improvement in reproductive outcome (subsequent pregnancy rate of 69% and live birth rate of 49% [48]) and a useful prophylactic procedure in primary infertile woman to improve the chance to achieving pregnancy [49]. It seems to be safer using hysteroscopic 5 Fr scissors than electrosurgery with a laser or resectoscope [48], because the use of electrosurgery may increase the risk of endometrial injury and uterine rupture during subsequent pregnancy compared with hysteroscopic scissors [50]. However, the role of uterine septum resection remains controversial. The literature describing an association between the septate uterus and infertility [20] or pregnancy loss consists almost exclusively of retrospective studies, and the only randomized, controlled trial failed to demonstrate any significant association [51]. The absence of any formal professional society recommendation in favor of septum resection supports the need for additional evidence. As already discussed regarding myomectomy, the necessity of an exhaustive and precise diagnosis before conducting an operative hysteroscopy, as part of the “see-and-treat” process, is also essential in the case of uterine septa. A correct diagnosis serves a dual purpose: it aids in ensuring therapeutic success and plays an essential role in averting possible complications. Despite the minimally invasive nature of operative hysteroscopy, it is important to acknowledge the critical need for the operator to have extensive surgical and clinical expertise. The alignment of an accurate diagnosis and proficient operator is the key factor in delivering the best patient outcomes and reducing potential risks. A dysmorphic uterus is a Müllerian anomaly that includes T-shaped and tubular-shaped infantilis uteri. Such malformations are associated with poor reproductive outcomes. Office hysteroscopy is considered a safe and effective procedure to expand the volume and normalize the appearance of the uterine cavity of dysmorphic uteri. Longitudinal lateral incisions are performed on the fibro-muscular constriction rings in the isthmic area of the uterine walls with a 5 Fr bipolar electrode or scissors [52].

## 3. Conclusions

In conclusion, office hysteroscopy offers several benefits including a shorter recovery time, reduced utilization of recovery rooms, and decreased reliance on general anesthesia. These advantages contribute to cost savings in various gynecological procedures. Therefore, it should be prioritized as the primary approach for managing intrauterine pathology. Traditional resectoscopic surgeries, on the other hand, should be reserved for more complex cases, such as endometrial ablation, or specific conditions like myomas with a diameter up to 1.5 cm, broad-base septa, or large-size polyps. Drawing from the lessons discussed, two essential take-home messages crystallize. First, the primacy of a correct diagnosis before undertaking an operative hysteroscopy is non-negotiable. Second, even though operative hysteroscopy is a minimally invasive procedure, it demands a high level of surgical and clinical experience. The operator’s acumen significantly impacts not only the success of the procedure but also the management of potential complications. Moreover, the world of outpatient hysteroscopy offers us an array of innovative procedures and tools, among which the new, high-cost, single-use technologies have received notable attention. While these technologies can significantly enhance the precision and efficacy of our procedures, the cost factor associated with them cannot be ignored. Therefore, a comprehensive understanding of these costs and their potential impact on the healthcare system and the patients is crucial. This balance between adopting new technologies and ensuring cost-effectiveness should remain a central theme in the evolution of outpatient hysteroscopy.

## Data Availability

Not applicable.
